# Wheat in the Mediterranean revisited – tetraploid wheat landraces assessed with elite bread wheat Single Nucleotide Polymorphism markers

**DOI:** 10.1186/1471-2156-15-54

**Published:** 2014-05-08

**Authors:** Hugo R Oliveira, Jenny Hagenblad, Matti W Leino, Fiona J Leigh, Diane L Lister, Leonor Penã-Chocarro, Martin K Jones

**Affiliations:** 1 IFM Biology, Linköping University, Linköping SE-581 83, Sweden; 2 CIBIO-Research Centre in Biodiversity and Genetic Resources, Campus Agrário de Vairão. R. Padre Armando Quintas, Vairão 4485-661, Portugal; 3 Nordiska Museet, Swedish Museum of Cultural History, Julita SE-643 98, Sweden; 4 The John Bingham Laboratory – National Institute for Agricultural Botany (NIAB), Huntingdon Road, Cambridge CB3 0LE, UK; 5 McDonald Institute for Archaeological Research, University of Cambridge, Downing Street, Cambridge CB2 3ER, UK; 6 Escuela Española de Historia y Arqueología en Roma-CSIC, Via di Torre Argentina 18, Roma 00186, Italy

**Keywords:** Ascertainment bias, Domestication, Linkage disequilibrium, Population structure, Single Nucleotide Polymorphism, *Triticum turgidum*

## Abstract

**Background:**

Single Nucleotide Polymorphism (SNP) panels recently developed for the assessment of genetic diversity in wheat are primarily based on elite varieties, mostly those of bread wheat. The usefulness of such SNP panels for studying wheat evolution and domestication has not yet been fully explored and ascertainment bias issues can potentially affect their applicability when studying landraces and tetraploid ancestors of bread wheat. We here evaluate whether population structure and evolutionary history can be assessed in tetraploid landrace wheats using SNP markers previously developed for the analysis of elite cultivars of hexaploid wheat.

**Results:**

We genotyped more than 100 tetraploid wheat landraces and wild emmer wheat accessions, some of which had previously been screened with SSR markers, for an existing SNP panel and obtained publically available genotypes for the same SNPs for hexaploid wheat varieties and landraces. Results showed that quantification of genetic diversity can be affected by ascertainment bias but that the effects of ascertainment bias can at least partly be alleviated by merging SNPs to haplotypes. Analyses of population structure and genetic differentiation show strong subdivision between the tetraploid wheat subspecies, except for durum and rivet that are not separable. A more detailed population structure of durum landraces could be obtained than with SSR markers. The results also suggest an emmer, rather than durum, ancestry of bread wheat and with gene flow from wild emmer.

**Conclusions:**

SNP markers developed for elite cultivars show great potential for inferring population structure and can address evolutionary questions in landrace wheat. Issues of marker genome specificity and mapping need, however, to be addressed. Ascertainment bias does not seem to interfere with the ability of a SNP marker system developed for elite bread wheat accessions to detect population structure in other types of wheat.

## Background

Bread wheat (*Triticum aestivum* ssp. *aestivum)* is, together with rice and maize, one of the main staple food crops of the world; in 2012 some 675 million tones were produced worldwide [1]. The importance of bread wheat has lead to the development of several species-specific genetic marker systems, such as SSRs (simple sequence repeats) [2], and DaRT markers [3] in addition to the generalist marker systems, for example AFLPs [4], previously in use. The development of Single Nucleotide Polymorphism (SNP) panels in wheat was long hampered by the lack of a reference genome sequence. However, the rapid development of sequencing methods recently enabled the completion of the wheat genome sequence [5], which has led to the development of SNP panels [6,7]. These genome-wide SNP panels allow not only wheat breeding to be addressed at a whole new level, but analysis of the evolution of domesticated wheat species can now be approached on a genomic scale.

The progenitor species of all domesticated wheat, wild emmer (*T. turgidum* ssp. *dicoccoides*), arose as an allotetraploid 300,000–500,000 years ago [8,9]. Much more recently, around 10,000 years ago, domesticated forms of emmer with a tough rachis emerged [10]. There is evidence that the two main cultivated, free-threshing wheats, tetraploid durum and hexaploid bread wheat, emerged from domesticated emmer wheat [11], although *de novo* domestication of durum from wild emmer has not yet been ruled out [12]. Bread wheat is believed to have arisen from a cross between a domesticated tetraploid wheat and the diploid wild grass *Aegilops tauschii*[8] although it is not clear whether the tetraploid ancestor was the naked durum or the hulled emmer [11,13,14].

Durum wheat (*T. turgidum* ssp. *durum*), the most widely grown tetraploid wheat, is cultivated to a far lesser extent than hexaploid bread wheat. The cultivation of other domesticated tetraploid wheats, such as emmer (*T. turgidum* ssp. *dicoccum*) and rivet (*T. turgidum* ssp. *turgidum*), is very limited. These relict crops have little agricultural importance, which has also lead to them being studied to a lesser extent than bread wheat. However, some SSR [15] and SNP [16] markers have been specifically developed for durum. The tetraploid wheats are an important genetic resource for breeding novel genetic diversity into bread wheat [17] and hence their genetic analysis is of importance. In addition, durum, emmer and rivet are an integral part of the evolutionary history of domesticated wheat. Exploring the distribution of genetic diversity in tetraploid wheats is thus valuable, both to document the genetic diversity present and to explore aspects of wheat evolution.

To date, the phylogeography of tetraploid wheat has mainly been explored in the Mediterranean region where its dispersal has been investigated using both AFLP and SSR markers [18,12]. Using the analysis of SSR markers in Italian emmer wheats, Isaac et al. [19] suggested a point of origin of emmer cultivation within the country; however, only a subset of the landrace accessions showed geographical structuring of genetic diversity. Oliveira et al. [12], also using SSR markers, showed that part of the genetic diversity found in durum wheats is geographically structured as an effect of the older evolutionary history of durum, but also that the effects of more recent seed trade could be detected through the wider dispersal of some genotypes.

The number of markers utilized in phylogeographic studies is a major component of the level of resolution that can be obtained [20]. For this reason, the potential number of markers and ease of genotyping make SNP markers an attractive choice for analyses of population structure when aiming to detect higher levels of genetic structuring. The rapid discovery of SNP markers in elite bread cultivars has provided a wealth of markers that also have the potential to be utilized in the genetic analysis of tetraploid wheats. There are, however, potential problems to the transfer of markers between genetically differentiated materials. Ascertainment bias, the selection of loci from a small number of individuals that are not representative of the different allele frequencies present in a population, not only underestimates biodiversity but can also affect analyses of population structure [21,22], although some authors have found limited effects on the general outcome [23]. However, there are few studies that compare the phylogeographic effects of biased and unbiased markers in the same set of individuals.

Here we revisit the study of tetraploid wheat landraces in the Mediterranean by Oliveira et al. [12]. An overlapping set of tetraploid wheat accessions is analysed using a panel of SNP markers in order to investigate whether a 15-fold increase in number of markers, although markers of a different type, will enable the detection of higher levels of phylogeographic structuring and further insight into the evolutionary history of tetraploid wheats.

## Methods

### Plant materials and SNP genotyping

Four durum wheat landraces (PI 52503, Cltr15472, PI 192483, TRI3055) and two rivet landraces (PI372456, TRI4082) were genotyped with 5386 SNPs in a panel developed by Allen et al. [24]. The landraces were chosen to represent distinct geographic regions and had previously been shown to belong to different ancestral populations based on their SSR genotypes [12]. DNA was extracted from the pooled first leaves of five individuals of each accession [12]. The accessions were assayed using the KBioscience Ltd. Competitive Allele Specific PCR SNP genotyping system, henceforth referred to as KASPar® (LGC Genomics Ltd., Hoddesdon, UK) [24].

Of the SNPs assayed, 444 SNPs produced clear calls in at least five of the six accessions and were chosen for further analysis (Additional file 1). These SNPs were used for genotyping an additional 99 accessions of tetraploid wheats (71 durum landrace accessions, seven rivets, eight wild emmers and 14 landrace emmer wheats; Additional file 2). DNA extraction and genotyping was carried out as above [12].

### Data analysis

Genetic diversity (calculated as Nei’s h, the expected heterozygosity under Hardy-Weinberg equilibrium) and Wright’s F_ST_ (the genetic differentiation between subpopulations) were estimated according to Nei [25] using purpose-written Perl scripts (available by request), as was Tajima’s D statistic [26]. For the calculation of F_ST_, significance values were determined by permutation tests (1000 permutations). Principal component analysis (PCA) was carried out with R software (R Development Core Team, 2007) using the *prcomp* command. In the PCA, data was analysed on an accession level where the number of copies of each allele at each locus were treated as independent variables.

Publicly available wheat SNP data for both tetraploid and hexaploid accessions [27], was added to our tetraploid wheat landraces data and utilized in analyses of population structure, using the model-based Bayesian clustering approach implemented in Structure [28]. Models assuming one (*K* = 1) to 20 (*K* = 20) clusters were used to test different datasets, using 50,000 MCMCs and 20,000 burn-in runs with the “admixture” model. Ten replicate runs were performed for each value of *K*. The best-fit model was determined by calculating *ΔK*[29] and from the similarity coefficients (H’ values) obtained from the software CLUMPP v 1.1.1 [30]. In CLUMPP the FullSearch algorithm was used for comparing runs with *K* < 4, whereas the Greedy algorithm was used for *K* = 4 to *K* = 6 and the LargeKGreedy algorithm for higher Ks. We also evaluated the “no admixture” model using the same settings. CLUMPP was further used to compare the output of Structure analysis of durum SNP and SSR data. Graphical representation of results of CLUMPP runs was obtained using the DISTRUCT software v 1.1 [31]. We also re-analysed part of the SSR data for durum generated by Oliveira et al. [12] (using the “admixture” model only). In this case accessions were treated as haploid as only single alleles had been scored for each accession; analyses were otherwise carried out as for the SNP data.

Linkage disequilibrium (LD), measured as D’ and r^2^, was calculated using purpose written Perl scripts. As phase could not be determined for accessions where both loci were heterozygous, such pairs were omitted from calculations of LD. For pairs of mapped SNPs (based on the preliminary wheat SNP genetic map [27]) map distance was used to explore the rate of decay in LD with distance. The *nls* command of the software R (R Development Core Team, 2007) was used to fit a non-linear regression line to the LD between pairs of linked SNPs in order to explore the decay of LD over distance.

## Results

### SNP validation and quality control

The full SNP panel developed by the Functional Genomics Group at the University of Bristol and collaborators [24] was assayed in six tetraploid landrace wheat accessions (durums Cltr15472, PI52503, PI192483 and TRI3055; rivets PI372456 and TRI4082) using the KASPar method (genotypes available at [27]). A total of 5386 SNPs were assayed of which 2714 (50.4%) were successfully genotyped in all six landraces.

Since these results were generated, many of the SNPs in the Allen et al. [24] panel have been mapped to the wheat genome [27]. As expected, the SNPs that had failed to genotype the initial six tetraploid accessions in many cases map to the D genome. However, 146 SNPs that mapped to the D genome in one of the two mapping panels used in [27], were successfully genotyped in at least one of the six tetraploids in the test panels. In addition, 83 SNPs mapping to the D genome were successfully genotyped in all six wheats in the test panel suggesting these markers have either been incorrectly mapped or genotype in more than one genome (Additional file 3).

Of the 5386 SNPs assayed in the six test individuals, 444 were chosen for further genotyping. These were genotyped in an additional 99 tetraploid wheats creating a dataset consisting of 105 tetraploid wheats (emmers, durums and rivets). Of the 444 SNPs assayed seven were later revealed to map to the D genome and were subsequently removed from further analyses (highlighted in red in Additional file 1).

Commercial varieties of durum can be considered to be pure lines and should therefore exhibit very limited heterozygosity, even when several individuals of the same accession have been pooled as in this study. Thus, as an additional quality control, we removed another 68 SNPs which exhibited heterozygosity in one or more of five commercial durum varieties included in this study (highlighted in red in Additional file 1). As the commercial durum varieties had a close common origin these were not used for further analysis. The final data set described below thus consisted of 100 tetraploid wheats genotyped for 369 SNP markers.

### Genetic diversity and evaluation of ascertainment bias effects

Of the accessions genotyped all but one, PI117420, exhibited heterozygosity at one or more loci. The number of heterozygous loci per accession ranged from zero to 84 with an average 17.8 heterozygous loci (4.8%, s.d. 20.2). This could be due to either variation among individuals within an accession (as DNA was extracted from bulks of five individuals), heterozygous individuals or a combination of the two. Among the different wheat types, durum and rivet exhibited more heterozygous loci (6.4 and 5.7% respectively) than landrace emmer and wild emmer (3.4 and 1.2% respectively) (Table 1).

**Table 1:** Average genetic diversity measures and Tajima’s D observed across all loci and linkage disequilibrium between unlinked markers for the different types of wheat studied

	**N**	**H**_**o**_^**a**^	**h**	**min h**^**b**^	**max h**^**c**^	**min h/max h**	**r**^**2e**^	**Tajima’s D**
Wild emmer	8	0.012	0.237	0.232	0.240	0.968		−2.739***
Landrace emmer	14	0.034	0.270	0.257	0.278	0.926		−0.586
Average for subsample of 7			0.231 (0.006)^d^	0.216 (0.006)	0.238 (0.006)	0.910		
Landrace durum	71	0.064	0.289	0.266	0.306	0.868	0.033; 0.014	1.419
Average for subsample of 7			0.251 (0.007)	0.226 (0.007)	0.267 (0.007)	0.848		
Landrace rivet	7	0.057	0.305	0.282	0.179	0.880		−1.594
All tetraploid wheats	99	0.056					0.036; 0.015	2.509*

^a^Average observed within accession heterozygosity.

^b^Minimum possible diversity for pooled individuals.

^c^Maximum possible diversity for pooled individuals.

^d^Average and standard deviation for 1000 replicates.

^e^Average and median linkage disequilibrium between unlinked markers.

*p < 0.05; ***p < 0.001.

Genetic diversity, measured as Nei’s h, was calculated from the SNP genotyping results. To account for the effects of pooling individuals, both the minimum and maximum genetic diversity possible were estimated in addition to the observed diversity (Table 1). The minimum diversity ranged from 86.8% of the maximum possible diversity in durum, to 96.8% of the maximum diversity in wild emmer; these differences in diversity measures could in some cases influence the outcome of comparisons between wheat subspecies.

The direct estimates of diversity (h in Table 1) were highest in rivet and durum landraces (0.305 and 0.289 respectively), followed by landrace emmer (0.270). The lowest diversity was detected in the wild emmers analysed (0.237). The high diversity of the durum and landrace emmer was in part caused by the higher number of accessions studied. Subsampling the durums and landrace emmers to the same sample size (N = 7) as rivets and wild emmer reduced the diversity from 0.289 to 0.251 for durums and from 0.270 to 0.231 for landrace emmers (Table 1).

The 369 SNPs used for genotyping were partly chosen on the basis of being variable in the panel of six test accessions, four durums and two rivets, and hence the high diversity of durum and rivet was most likely also caused by ascertainment bias. To quantify this effect we compared genetic diversity in the six test accessions using the data for the complete 5386 SNP panel and the subset of 369 SNPs used in the analysis of the remaining accessions. The average diversity for the test panel across the 5386 SNPs of the full panel was only 0.083 compared to 0.328 for the 369 SNPs in the final dataset, or 25.3% of the diversity in the final assay panel. Looking only at the 2714 SNPs that successfully amplified in all the test accessions the genetic diversity (0.164) was still only 50.2% of that of the 369 SNPs used for the extended set of accessions.

It has been suggested that the effects of ascertainment bias can be alleviated by combining SNPs into haplotypes [32]. To investigate this we combined neighbouring SNPs in two datasets (the six test individuals genotyped for 5368 and 369 SNPs, respectively) into non-overlapping haplotypes two to ten bases long and recalculated the genetic diversity based on haplotypes. That is, for making two-SNP haplotypes the first two SNPs along a chromosome were combined to create the first haplotype marker, then the following two SNPs were combined to create the second haplotype marker and so on. While the diversity based of the unbiased 5386 SNP set approached that of the biased 369 SNP set, going from 53.2% (0.215 vs 0.412 for the 5386 and 369 SNP set respectively) at the two-SNP haplotype stage to 65.8% (0.466 vs 0.708) at the ten-SNP haplotype stage, the genetic diversity was still significantly lower for the unbiased haplotypes based on the 5386 SNP set also at the ten-SNP haplotype stage (one-tailed t-test, p < 0.01).

### Linkage disequilibrium in tetraploid wheats and durum landraces

LD was measured both as D’ and r^2^. As both measures gave similar results only the latter is reported below. For pairs of unlinked loci (only pairs of loci on different chromosomes were used) the r^2^ values for most pairs showed fairly low levels of LD with a skewed tail of higher values of LD (Additional file 4). Across all tetraploid wheats the median r^2^ for pairs of unlinked loci was 0.015 (average 0.036, Table 1). The durum accessions showed slightly but significantly less LD than the combined set of all tetraploid wheats with a median r^2^ of 0.014 (average 0.033, Table 1, two-tailed t-test: p < < 0.001). However, r^2^ values higher than 0.3 were more common among durums than when considering all tetraploid wheats together. For both tetraploid wheats in general, and to a larger extent for the landrace durum wheats, a number of pairs of loci (7 and 24 respectively), located on different chromosomes but in complete LD was detected.

**Figure 1:**
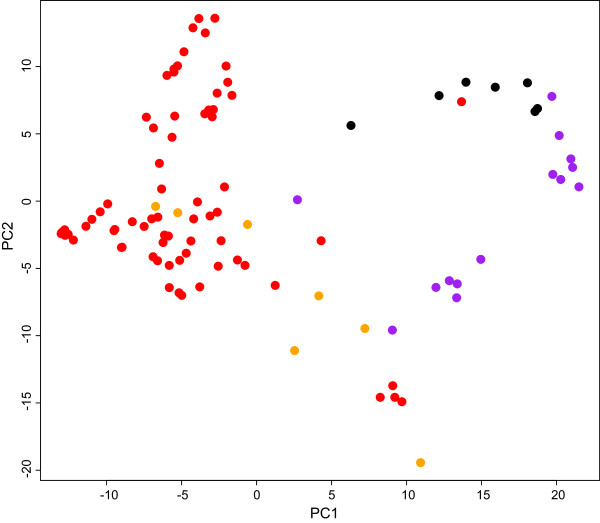
**Linkage disequilibrium (r**^**2**^**) between linked markers, located 20 cM or less apart, plotted against the genetic distance with a non-linear regression line fitted to the values. a)** All tetraploid wheats; **b)** durum landraces only.

By fitting a non-linear regression to the LD for pairs of loci located on the same chromosome, we noted that LD quickly decayed to background levels over a distance of less than 10 cM for tetraploid wheats in general, whereas for the durum landraces only LD decayed to background levels somewhat slower, over a distance of less than 15 cM (Figure 1). However, in both datasets pairs of loci with r^2^ of more than 0.4 could be detected over several tens of cM (Additional file 5a, b).

LD measured between neighbouring loci across all tetraploid wheats identified a few regions of high LD. Using 0.3 as an arbitrary cut-off, chromosomes 1A, 1B, 3A and 5B all contained regions where 3 or more neighbouring pairs had high LD, covering regions of up to 15 cM.

### Distribution of genetic diversity in tetraploid wheats

We calculated Tajima’s D statistic across all loci for the complete data set and for the different types of wheat. The complete dataset showed a significantly positive Tajima’s D and evidence of population subdivision. In contrast, within type values were all non-significant with the exception of wild emmer, which had a significantly negative value of Tajima’s D, indicative of population growth (Table 1). The landrace durum was the only type of wheat showing a positive, albeit non-significant, Tajima’s D.

**Figure 2:**
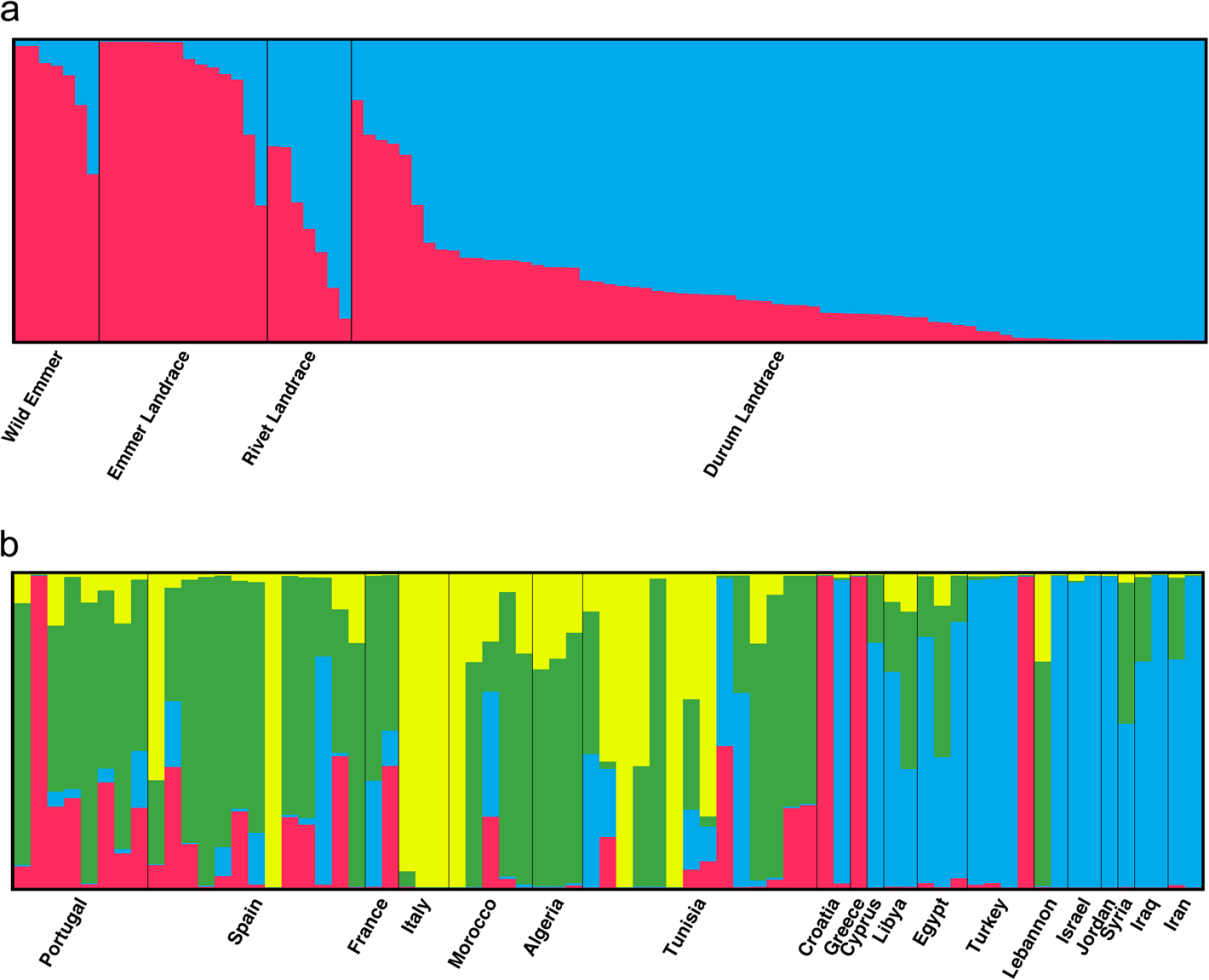
**Results of Principal Component Analysis (PCA) of tetraploid wheat accessions based on 369 SNP markers.** Each dot represents the location of a wheat accession along the first two principal components. Black = wild emmer; purple = landrace emmer; red = durum; orange = rivet.

Pairwise F_ST_ values between the different types of wheat were all highly significant and ranged between 0.033, comparing durum and rivet, and 0.214 when comparing wild emmer and rivet (Table 2). This was partly reflected in the PCA of the different accessions where the first two PCs explained 9.85 and 6.90% of the variation, respectively. Rivets (orange in Figure 2) primarily clustered among the durums (red) along PC1 and to a greater extent PC2, while there was a larger level of separation between emmers (black and purple) and durums along the first PC and between wild and domesticated emmers along PC2 (black and purple respectively). No type of wheat was, however, uniquely separated from the others along the two first PCs (Figure 2).

**Table 2:** **Pairwise F**_**ST **_**values between pairs of wheat types**

	**Wild emmer**	**Landrace emmer**	**Landrace durum**
Landrace emmer	0.146	-	-
Landrace durum	0.091	0.112	-
Landrace rivet	0.214	0.167	0.033

All values are significant values at p < 0.001.

For all our Structure analyses of SNPs we tested both the “admixture” and the “no admixture” model. The two models in general showed a good agreement in the number of clusters for which the highest support was obtained. Although there were some differences regarding individual proportional memberships to the different clusters (Additional file 6), the two models yielded the same general conclusions. The “admixture” model produced results that seemed more informative about gene flow between the different groups and we thus based our main analysis on these results.

**Figure 3:**
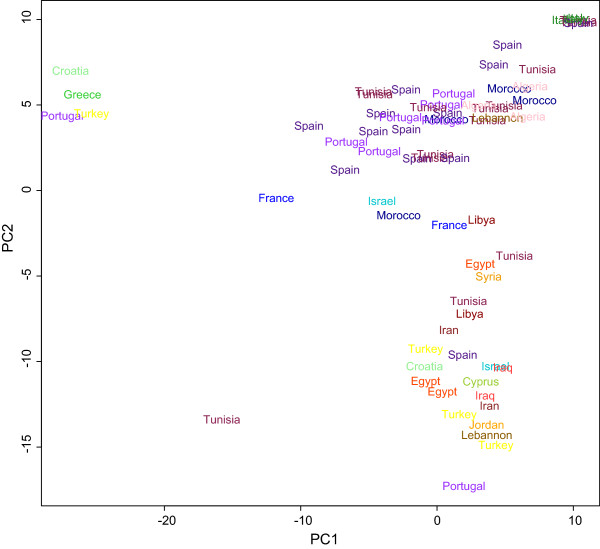
**Structure plot of a) tetraploid wheats in the *K =*** **2 model and b) durums only in the *K =*** **4 model.** Each accession is depicted by a vertical line segmented into *K* coloured sections. The length of each section is proportional to the estimated membership coefficient (Q) of the accession to each one of the *K* number of clusters. Accessions are assembled by a) taxon and b) country of origin.

Similar results to the PCA of tetraploid wheats were obtained from Structure analysis where both *ΔK* and CLUMPP H’ values suggested *K* = 2 as the most likely clustering, but also with support also for *K* = 4 from Δ*K* and *K* = 5 from CLUMPP H’ values. At *K* = 2 the tetraploid wheats were primarily split into one group consisting of wild and landrace emmers and one group comprising durums and rivets (Figure 3a) but with mixed ancestry of many accessions. The emmer wheats together remained a distinct cluster also at higher levels of *K*. Instead a mixed ancestry was introduced for the durums and rivets, but no cluster unique for each type could be detected (Additional file 7).

**Figure 4:**
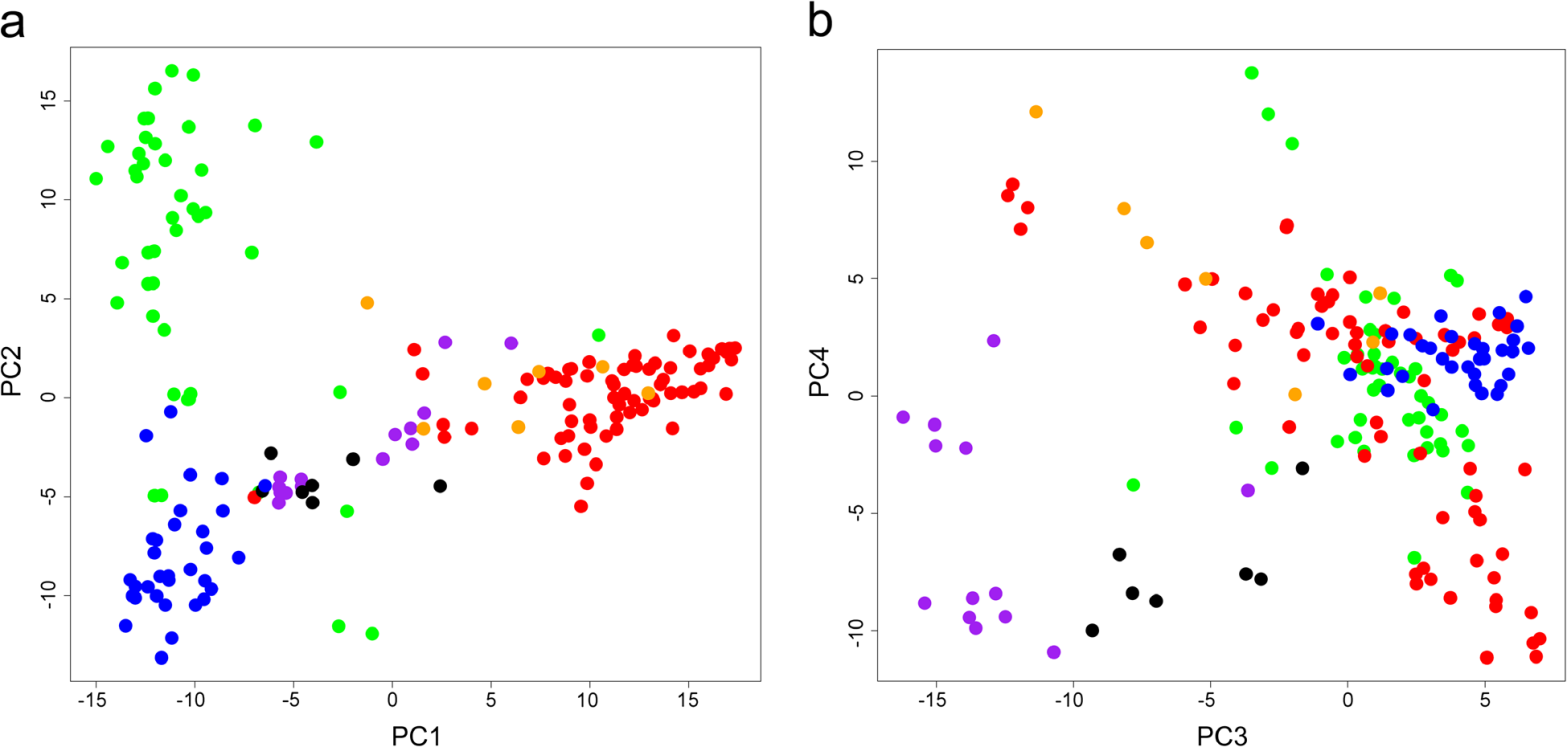
**Results of Principal Component Analysis of durum wheat landrace accessions coloured and labelled by country of origin.** PCA was based on the allele frequencies of 369 SNP markers.

Geographic structuring was also explored for the durums alone. When analysing the data with the software Structure, the computation of *ΔK* suggested the distribution of genetic diversity was best described by four clusters (three for the “no admixture” model) while CLUMPP H’ values were similarly high for *K* = 2 to *K* = 4 (highest for *K* = 3 for the “no admixture” model). Neither the PCA nor any level of clustering showed a strong geographic structure. Instead, different accessions from the same country or region clustered together already at *K* = 2 in the Structure analysis (Additional file 8a). Some geographical patterns could, however, be detected. At *K* = 4 (Figure 3b) an eastern group (blue in Figure 3b) contained accessions from Cyprus, Croatia, Egypt, Iran, Iraq, Israel, Jordan, Lebanon and Turkey. A western group (green) contained accessions from Algeria, France, Morocco, Portugal, Spain and Tunisia. A second mainly eastern cluster (red) contained other accessions from Croatia and Turkey, but also from Greece and one Portuguese accession. The fourth cluster contained all Italian accessions, and also accessions from Spain and Tunisia (yellow). Similar clustering could be detected in the PCA (Figure 4).

### Distribution and structuring of genetic diversity between ploidy levels

In addition to the tetraploid accessions genotyped in this study, the publicly available genotypes for the same SNPs in bread wheats were obtained [27]. These genotypes, derived from 36 winter-sown and 12 spring-sown commercial bread wheats and 32 landrace bread wheats (23 winter and 9 spring, respectively, Additional file 2), allowed us to explore the distribution of genetic diversity between tetraploid and hexaploid wheats.

**Figure 5:**
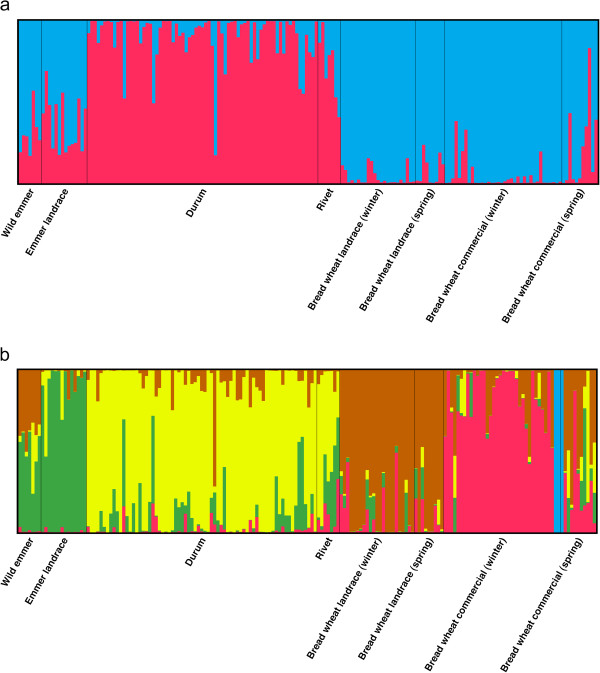
**Results of Principal Component Analysis of all wheats (excluding Yumai 34, Anahuac 75 and Ukrainka 3).** Black = wild emmer; purple = landrace emmer; red = durum; orange = rivet; blue = landrace bread wheat; green = commercial bread wheat **a)** First and second PCs, **b)** third and fourth PCs.

A PCA of all 184 accessions widely separated three bread wheats (Yumai 34, Anahuac 75 and Ukrainka 3) from all other accessions along the first PC (Additional file 9). These three accessions were consequently excluded from all further analysis. For the remaining 181 accessions, the first two PCs explained 10.83 and 6.48% of the variation, respectively, and individuals showed a high degree of clustering according to type (Figure 5a). Wild and domesticated emmer accessions (black and purple respectively in Figure 5) were centrally located along the first PC where hexaploid wheats (blue and green) and durums and rivets (red and orange respectively) were to a large degree separated from emmers and each other. The second PC primarily separated landrace bread wheats from commercial bread varieties. The third PC explained only 5.71% of the variation but added separation between wild and domesticated emmer (Figure 5b). No clear separation between rivets and durums could be detected along any of the first four PCs.

**Figure 6:**
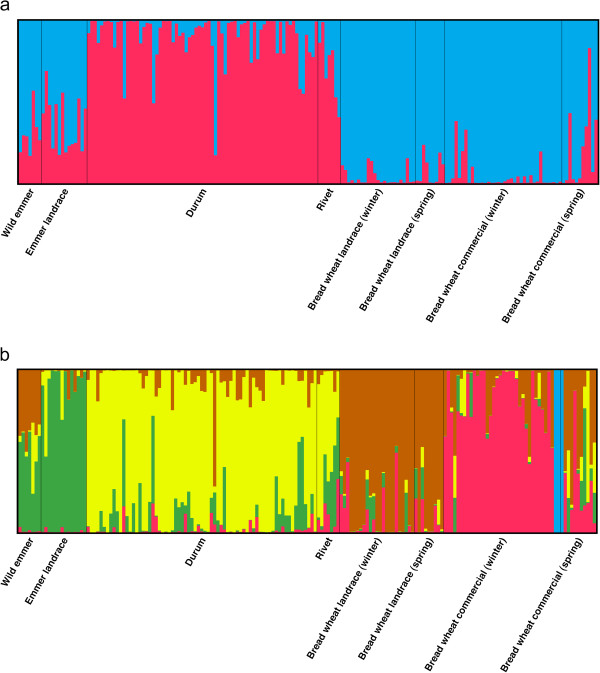
**Results of Structure analysis of the complete set of wheat accessions based on 369 SNPs for a) *K*** **= 2 model, b) *K*** **= 5 model.**

Structure analysis of all wheat types was carried out to further explore genetic differences between different types. At *K* = 2 durum and rivet accessions mainly clustered together while the bread wheats were grouped in a different cluster and emmer wild emmer accessions showed a mixed ancestry (Figure 6a). At *K* = 3 the commercial winter bread wheats clustered away from the commercial spring and landrace bread wheats and *K* = 4 separated the outlying wheats from the PCA. *K* = 5 saw the formation of an emmer cluster (Figure 6b). Higher levels of K, although supported by *ΔK* values (supporting *K* = 2 and *K* = 8) and CLUMMP H’ values (supporting *K* = 2 and *K* = 9), mainly introduced mixed ancestry to the accessions (Additional file 10a and b).

### Comparisons between different marker systems

The durum accessions SNP genotyped in this study had previously been genotyped for 29 SSR markers [12], which allowed direct comparison of the two marker systems. The multi-allelic SSR markers (average 11.2 alleles per marker) had higher levels of genetic diversity (average 0.654 vs. 0.289, one-tailed t-test, p < 0.001). However, upon merging neighbouring SNPs to haplotypes, already at the two SNP haplotype stage, the genetic diversity of the haplotypes (average 0.625) was as high as that of the SSRs (one-tailed t-test, p = 0.158).

We also analysed the durum accessions present in both the Oliveira et al. [12] and the present datasets with PCA and Structure analysis to compare the ability of the two marker systems to detect genetic structure. For the SNP dataset the ΔK and CLUMPP H’ values both suggested structure was best described by four clusters, while for the SSR dataset ΔK and CLUMPP H’ were both highest at *K* = 2, suggesting that the SNP markers were able to detect higher levels of structuring than the SSRs.

Many accessions, such as most of those included in the Italian cluster described above, clearly demonstrated similar clustering using the two marker systems; however, this was not the case for all accessions (Additional file 8). Comparing the outcome of the Structure analysis of the two marker systems using CLUMPP, it was clear from the H’ values that the correspondence between the two different marker systems was markedly lower than between repeated Structure runs of the same marker data (e.g. 0.996 and 0.998 for ten runs of SSRs and SNP markers respectively at *K* = 2, vs 0.776 comparing SNPs and SSRs). The degree to which accessions were assigned to a Structure cluster was highly correlated at *K* = 2 (*r* = 0.797, p < < 0.001) but with marked differences for some accessions. For example, the accession CItr15519 was assigned almost completely to one group based on SNP markers, but to the opposite group based on SSRs. A PCA of the SSR data showed lower explanatory power of the two first PCs (6.0 and 4.4% for SSRs compared to 8.3 and 7.8% for SNPs).

## Discussion

### Marker transferability between wheat types

Once developed, SNPs in crops constitute amenable high-throughput genetic markers, sufficiently abundant to be useful for many applications in plant breeding as well as in explorations of crop evolutionary history. The markers developed for such SNP panels are, however, a direct result of the individuals chosen for identifying genetic diversity in the first place. This can lead to an ascertainment bias in estimates of genetic diversity, since alleles segregating at low frequencies will be disregarded.

Ascertainment bias will, in particular, be an issue when the markers are used to compare those populations used to develop them with other populations [33]. In this study, the use of a test panel of four durums and two rivet accessions to identify polymorphic loci in tetraploid wheats allowed the quantification of the effect of ascertainment bias on marker selection. The markers eventually used showed a genetic diversity in the test panel that was twice that of all the successfully amplifying markers. The increasing levels of genetic diversity found in this study, going from wild emmer to landrace emmer to durum and rivet, is in contrast to what has been shown using other marker systems [14,11,34], and may well be ascribable to ascertainment bias. This is particularly evident in the number of heterozygous loci which one would expect to be higher in wild emmer and emmer than in durum landraces, due to their higher rates of outbreeding, older evolutionary history and lower selection for desired agronomic traits. In fact the number of heterozygous loci are actually lower in the emmers. The choice of markers initially found to be polymorphic in durum landraces biased the levels of polymorphism towards the latter, making them appear to have higher diversity than wild and cultivated emmer. Correcting for sample size did reduce the genetic diversity of landrace emmer and durum, demonstrating a partial effect of sample size; however, rivet and durum remained the most diverse wheat types. It has been suggested that ascertainment bias can be circumvented by combining SNPs into haplotypes [32]. Combining SNPs into haplotypes certainly reduced the effects of ascertainment bias on estimating genetic diversity, but it was not able to fully compensate for it.

Several of the accessions amplified more than one allele at 10% or more of the markers. Such high levels of heterozygosity are not expected from a self-fertilising plant and thus most likely constitute within-accession diversity. The SNP genotyping system used does not reliably allow quantification of allele frequencies in pooled samples and our estimates show that the differences between maximum and minimum possible diversity can be more than 10% in a dataset such as the one presented here. Bulked samples does allow cost-efficient capture of genetic diversity in heterogenous landraces or wild populations, but separate analyses of several individuals from each accession would be required to more accurately determine their genetic composition (see e.g. Forsberg et al., forthcoming).

### Mapping of markers and linkage disequilibrium

Seventy-one SNPs mapping to the D genome were successfully genotyped in the test panel of six tetraploid wheats, where only the A and B genomes occur. This is a cause of concern as it suggests some markers may have been incorrectly mapped or are not genome specific. To minimize the number of such markers, we excluded all markers mapping to the D genome. We also removed putative cross-amplifying markers showing within-accession heterozygosity in five genotyped improved durum varieties, since these accessions can be expected to be genetically monomorphic. High heterozygosity in SNP loci in wheat lines selfed for many generations has also been noted by Würschum et al. [35] as an unexpected and yet frequent occurrence; this lead to their removal of all SNPs showing heterozygosity in more than 30% of the lines studied. Clearly, information about genome specificity and map-position of SNP markers in the wheat genome needs to be critically evaluated. As SNP genotyping technology improve further, genome specific markers will be more common and strategies to produce these have already been developed [7,24].

Our background level of LD was lower than that previously detected in bread wheat using other marker systems, but with LD between linked loci decaying on a similar scale as in previous studies of bread wheat [35-38] and durum [39,40], and slower than what was demonstrated in both bread wheat and durum by Somers et al. [41]. For association mapping of traits segregating in Mediterranean landrace durum, assuming a ratio of mapping to physical distance of 1 cM/Mb, a marker density of several thousand markers is required to capture haplotype blocks across the genome. We found, however, as has been shown in previous studies of wheat, that LD varies between different chromosomes and chromosomal regions [35,37], which will affect local resolution in association mapping.

### Insights into the evolutionary history of domesticated wheat

Population structure studies based on SNP data can potentially be affected by ascertainment bias when the SNP panel used has been developed for populations or species other than those analysed [21,22]. Hübner et al. [23] used unbiased SSRs and SNPs that were developed for elite barley (thus being biased markers), to investigate population structure in the same populations of wild and domesticated barley. They detected an underrepresentation of rare alleles when analysing the genetic diversity of wild barley with the SNPs developed for elite breeds, which is a result of ascertainment bias. Nevertheless the authors found that the two marker systems detected the same population structure and number of clusters for the wild barley populations, suggesting that the effect of ascertainment bias on detection of genetic structuring was minor.

In this study we find strong support for independent evolutionary trajectories for tetraploid and hexaploid wheats. Structure analysis first separates hexaploid wheats from durums and rivets, with intermediate clustering of wild and landrace emmers (Figure 6a) as would be expected if the latter are the ancestors of both durum and bread wheat. In the PCA shown in Figure 5a, wild emmer accessions are located in the centre of the plot, as would also be expected from an ancestral gene pool. Clustering with the wild emmer is domesticated emmer and the gradual development of durums is observed along the first PC. At the other end of the first PC, landrace bread wheats cluster away from the wild emmers in the hexaploid domestication path. The clear separation of durums and bread wheats, with an intermediate position of emmers, supports the suggested emmer, rather than durum, ancestry of bread wheat [11,13,14].

More surprisingly, along the third PC, wild emmer assumes an intermediate location compared to bread wheats and domesticated emmer landraces, tentatively suggesting gene flow from wild emmer into the progenitor of hexaploid wheat or directly to the A and B genomes of hexaploid wheat. The location of durum along the third PC likewise suggests a role of wild emmer in the formation of the durum gene pool. Further studies directly targeted at clarifying the evolutionary origin of domesticated wheat and the role of gene flow between different types of wheat are needed.

### Taxonomy and structure of tetraploid wheats

Durum and rivet have traditionally been classified as different taxa, based on different ear morphology and by the latter’s broader tolerance to moist and cold environments. Neither the PCAs presented here nor the Structure analysis separate durum and rivet from each other. The F_ST_ value between the two was the lowest of the pairwise F_ST_ values calculated (albeit significant). As in Oliveira et al. [12] no genetic support for separating the two into different taxa was found. We thus conclude that gene flow between durum and rivet is probably frequent enough to prevent them from becoming genetically distinct and they should be classified as the same taxon.

Given the number of markers used in this study it is unlikely that our genome-wide values of Tajima’s D are caused by selection acting on all the different markers. More likely they reflect components of population history that affect the whole genome equally, such as population subdivision or population growth for example following a bottleneck. Ascertainment bias resulting from the marker selection in this study should lead to an underrepresentation of rare alleles [23]. As a lack of rare alleles will result in a positive value of Tajima’s D, ascertainment bias should act to increase estimates of D. In spite of this, only durum (the type least affected by ascertainment bias) had a positive D and wild emmer had a significantly negative D. The negative D of wild emmer is surprising as it suggests population growth, which would be expected to be more significant in the domesticated wheat types. However, this result corroborates that of Haudry et al. [14] and tentatively suggests a past bottleneck in wild emmer.

Although the Tajima’s D of landrace durum was not significantly positive, it was markedly higher than those of the other types of wheat. This, together with the somewhat higher LD found in durum wheats compared to the complete set of tetraploid wheats, suggests that a certain level of population subdivision is present in the landrace durum analysed here, possibly resulting in the observed genetic structuring. The increased number of markers allowed for the detection of higher levels of genetic structuring than those found by Oliveira et al. [12] (four vs. two). As in Oliveira et al. [12], geographically isolated durum populations were not found, although broad scale structuring patterns could be detected.

### Comparison of marker systems

Most of the tetraploid wheats used in this study had previously been genotyped for 29 SSR markers [12]. This enabled the direct comparison of the two marker systems used. SSR markers show a higher diversity than each single SNP marker, which is in agreement with earlier studies [35,42]. Nevertheless, when merging as few as two neighbouring SNPs, the haplotypes show as much genetic diversity as the SSR markers. For capturing genetic diversity, two-SNP haplotype markers can thus be as efficient as an SSR. It is, however, worth noting that in the setup used, only a single allele could be scored for each SSR marker in each accession, while heterozygosity could be detected for the SNP markers.

Haasl and Payseur [43], using simulated datasets, calculated that 1000 ascertained SNPs were required to equal the performance of 100 non-ascertained SSRs in inferring correct population structure. In the study described here, the first two PCs of a PCA of the SNP markers explained a larger proportion of the genetic diversity, and the 15-fold increase in marker number did allow the discernment of higher levels of clustering for durum wheat. Although the correspondence in clustering between the two marker systems was not complete and some accessions clustered differently in the PCA and Structure analysis, the general conclusions drawn from the two marker systems remained the same.

## Conclusion

The use of SNPs in determining population structure in wheat species shows promise. The SNPs used here were discovered in a panel of elite cultivars, which likely reduces the capacity to compare genetic diversity between accessions from other ploidy level or subspecies; however this does not appear to invalidate the usefulness of the method for some purposes. Ascertainment bias does not seem to interfere with the ability of a SNP marker system developed for elite bread wheat to detect population structure in other types of wheat. More SNP markers with greater genome specificity and better mapping data will improve the resolution of this approach.

### Availability of supporting data

The data set supporting the results of this article is included or referred to within the article and its additional files.

## Abbreviations

AFLP: Amplified-fragment-length polymorphism; cM: Centi-Morgan; FST: Fixation index; LD: Linkage disequilibrium; Mb: Mega-base-pair; PC: Principal component; PCA: Principal component analysis; PCR: Polymerase chain reaction; SNP: Single Nucleotide Polymorphism; SSR: Simple sequence repeat.

## Competing interests

The authors declare that they have no competing interests.

## Authors’ contributions

JH analysed the data and wrote the manuscript. HRO designed the experiment, carried out laboratory work and contributed to writing the manuscript and data analysis. MWL contributed to manuscript writing and data analysis. FJL, DLL. LP-C and MKJ contributed to experimental design, discussion and manuscript preparation. All authors read and approved the final manuscript.

## Supplementary Material

Additional file 1 **SNPs used (primers, mapping and Blast information retrieved from [**[27]**]).** Markers that were removed before the final analysis are highlighted in red. Click here for file

Additional file 2 **Accessions used in this study.** Tetraploid landrace accessions were originally screened for this work. The remaining accessions were screened by the Crop Improvement Research Club, as described in [27] and in [24] and genotypes for these accessions is publically available at these references. Click here for file

Additional file 3 List of SNPs mapping to the D genome that successfully amplified in some or all of the tetraploid test panel accessions described in this work. Click here for file

Additional file 4 **Distribution of linkage disequilibrium values calculated between pairs of loci located on different chromosomes.** a) All values of r^2^, b) Values of r^2^ from 0.2 and higher. Click here for file

Additional file 5 **Linkage disequilibrium (r**^**2**^**) between linked markers plotted against genetic distance with a non-linear regression line fitted to the values.** a) All tetraploid wheats; b) all durum landraces. Click here for file

Additional file 6 **Results of Structure analysis of wheat accessions based on 369 SNPs using the “*admixture*” option (top panel) and the “ *no admixture*” option (bottom panel) for a) *K* = 2 model with the durum accessions; b) *K* = 4 model with durum accessions; c) *K* = 2 model with the complete set of accessions; d) *K* = 5 model with the complete set of accessions.** Click here for file

Additional file 7 **Results of Structure analysis of the *K =*** **3 model for the tetraploid wheat set.** Click here for file

Additional file 8 **Results of Structure analysis of the set of durum accessions for the *K =* 2 model based on a) 369 SNPs and b) 29 SSRs respectively.** Click here for file

Additional file 9 **Results of Principal Component Analysis of the complete set of wheat accessions.** The distinctiveness of Yumai 34, Anahuac 75 and Ukrainka is evident (top left corner). Black = wild emmer; purple = landrace emmer; red = durum; orange = rivet; blue = landrace bread wheat; green = commercial bread wheat. Click here for file

Additional file 10 **Results of Structure analysis of the complete set of wheat accessions for the a) *K =* 8 model and b) *K =* 9 model.** Click here for file

## References

[B1] The US Department of Agriculture Foreign Agricultural Service http://www.pecad.fas.usda.gov/highlights/2010/11/global%20durum/

[B2] Röder MS Korzun V Wendehake K Plaschke J Tixier MH Leroy P Ganal MW A microsatellite map of wheat Genetics 2009 149 4 2007 2023 9691054 10.1093/genetics/149.4.2007 PMC1460256

[B3] Akbari M Wenzl P Caig V Carling J Xia L Yang S Uszynski G Mohler V Lehmensiek A Kuchel H Hayden MJ Howes N Sharp P Vaughan P Rathmell B Huttner E Kilian A Diversity arrays technology (DArT) for high-throughput profiling of the hexaploid wheat genome Theor Appl Genet 2006 113 8 1409 1420 10.1007/s00122-006-0365-4 17033786

[B4] Huang X Zeller FJ Hsam SL Wenzel G Mohler V Chromosomal location of AFLP markers in common wheat utilizing nulli-tetrasomic stocks Genome 2000 43 2 298 305 10.1139/g99-118 10791818

[B5] Brenchley R Spannagl M Pfeifer M Barker GL D'Amore R Allen AM McKenzie N Kramer M Kerhornou A Bolser D Kay S Waite D Trick M Bancroft I Gu Y Huo N Luo MC Sehgal S Gill B Kianian S Anderson O Kersey P Dvorak J McCombie WR Hall A Mayer KF Edwards KJ Bevan MW Hall N Analysis of the bread wheat genome using whole-genome shotgun sequencing Nature 2012 491 7426 705 710 10.1038/nature11650 23192148 PMC3510651

[B6] Cavanagh CR Chao S Wang S Huang BE Stephen S Kiani S Forrest K Saintenac C Brown-Guedira GL Akhunova A See D Bai G Pumphrey M Tomar L Wong D Kong S Reynolds M da Silva ML Bockelman H Talbert L Anderson JA Dreisigacker S Baenziger S Carter A Korzun V Morrell PL Dubcovsky J Morell MK Sorrells ME Hayden MJ Genome-wide comparative diversity uncovers multiple targets of selection for improvement in hexaploid wheat landraces and cultivars Proc Natl Acad Sci U S A 2013 110 20 8057 8062 10.1073/pnas.1217133110 23630259 PMC3657823

[B7] Akhunov ED Nicolet C Dvorak J Single nucleotide polymorphism genotyping in polyploid wheat with IlluminaGoldenGate assay Theor Appl Genet 2009 119 507 517 10.1007/s00122-009-1059-5 19449174 PMC2715469

[B8] Peng JH Sun D Nevo E Domestication evolution, genetics and genomics in wheat Mol Breed 2011 28 3 281 301 10.1007/s11032-011-9608-4

[B9] Zohary D Hopf M Weiss E Domestication of Plants in the Old World 2012 3 Oxford: Oxford University Press

[B10] Salamini F Ozkan H Brandolini A Schäfer-Pregl R Martin W Genetics and geography of wild cereal domestication in the near east Nat Rev Genet 2002 3 6 429 441 12042770 10.1038/nrg817

[B11] Luo MC You FM Kawahara T Waines JG Dvorak J The structure of wild and domesticated emmer wheat populations, gene flow between them, and the site of emmer domestication Theor Appl Genet 2007 114 947 959 10.1007/s00122-006-0474-0 17318496

[B12] Oliveira HR Campana M Jones H Hunt H Leigh F Lister DL Jones MK Tetraploid wheat landraces in the Mediterranean basin: taxonomy, evolution and genetic diversity PLoS ONE 2012 7 5 e37063 10.1371/journal.pone.0037063 22615891 PMC3353906

[B13] Dvorak J Deal KR Luo MC You FM von Borstel K Dehghani H The origin of spelt and free-threshing hexaploid wheat J Hered 2012 103 3 426 441 10.1093/jhered/esr152 22378960

[B14] Haudry A Cenci A Ravel C Bataillon T Brunel D Poncet C Hochu I Poirier S Santoni S Glémin S David J Grinding up wheat: a massive loss of nucleotide diversity since domestication Mol Biol Evol 2007 24 1506 1517 10.1093/molbev/msm077 17443011

[B15] Korzun V Röder MS Wendehake K Pasqualone A Lotti C Ganal MW Blanco A Integration of dinucleotide microsatellites from hexaploid bread wheat into a genetic linkage map of durum wheat Theor Appl Genet 1999 98 8 1202 1207 10.1007/s001220051185

[B16] Trebbi D Maccaferri M de Heer P Sørensen A Giuliani S Salvi S Sanguineti MC Massi A van der Vossen EA Tuberosa R High-throughput SNP discovery and genotyping in durum wheat (*Triticum durum* Desf.) Theor Appl Genet 2011 123 4 555 569 10.1007/s00122-011-1607-7 21611761

[B17] Jauhar PP Damania AB Damania AB Alien gene transfer and genetic enrichment of bread wheat Biodiversity and wheat improvement 1993 New York: John Wiley & Sons 103 119

[B18] Moragues M Moralejo M Sorrels M Royo C Dispersal of durum wheat [Triticum turgidum L. ssp. turgidum convar. durum (Desf.) MacKey] landraces across the Mediterranean basin assessed by AFLPs and microsatellites Genet Resour Crop Evol 2007 54 1133 1144 10.1007/s10722-006-9005-8

[B19] Isaac AD Muldoon M Brown KA Brown TA Genetic analysis of wheat landraces enables the location of the first agricultural sites in Italy to be identified JArch Sci 2010 37 950 956

[B20] Nordborg M Hu TT Ishino Y Jhaveri J Toomajian C Zheng H Bakker E Calabrese P Gladstone J Goyal R Jakobsson M Kim S Morozov Y Padhukasahasram B Plagnol V Rosenberg NA Shah C Wall JD Wang J Zhao K Kalbfleisch T Schulz V Kreitman M Bergelson J The pattern of polymorphism in Arabidopsis thaliana PLoS Biol 2005 3 7 e196 10.1371/journal.pbio.0030196 15907155 PMC1135296

[B21] Russell J Dawson IK Flavell AJ Steffenson B Weltzien E Booth A Ceccarelli S Grando S Waugh R Analysis of >1000 single nucleotide polymorphisms in geographically matched samples of landrace and wild barley indicates secondary contact and chromosome-level differences in diversity around domestication genes New Phytol 2011 191 2 564 578 10.1111/j.1469-8137.2011.03704.x 21443695

[B22] Albrechtsen A Nielsen FC Nielsen R Ascertainment biases in SNP chips affect measures of population divergence Mol Biol Evol 2010 27 11 2534 2547 10.1093/molbev/msq148 20558595 PMC3107607

[B23] Hübner S Günther T Flavell A Fridman E Graner A Korol A Schmid KJ Islands and streams: clusters and gene flow in wild barley populations from the Levant Mol Ecol 2012 21 5 1115 1129 10.1111/j.1365-294X.2011.05434.x 22256891

[B24] Allen AM Barker GL Berry ST Coghill JA Gwilliam R Kirby S Robinson P Brenchley RC D'Amore R McKenzie N Waite D Hall A Bevan M Hall N Edwards KJ Transcript-specific, single-nucleotide polymorphism discovery and linkage analysis in hexaploid bread wheat (*Triticum aestivum* L.) Plant Biotechnol J 2011 9 9 1086 1099 10.1111/j.1467-7652.2011.00628.x 21627760

[B25] Nei M Analysis of gene diversity in subdivided populations Proc Natl Acad Sci U S A 1973 70 3321 3323 10.1073/pnas.70.12.3321 4519626 PMC427228

[B26] Tajima F Statistical method for testing the neutral mutation hypothesis by DNA polymorphism Genetics 1989 123 3 585 595 2513255 10.1093/genetics/123.3.585 PMC1203831

[B27] Cereals Database UK http://www.cerealsdb.uk.net/cerealgenomics/CerealsDB/indexNEW.php

[B28] Pritchard JK Stephens M Donnelly P Inference of population structure using multilocus genotype data Genetics 2000 155 945 949 10835412 10.1093/genetics/155.2.945 PMC1461096

[B29] Evanno G Regnaut S Goudet J Detecting the number of clusters of individuals using the software STRUCTURE: a simulation study Mol Ecol 2005 14 2611 2620 10.1111/j.1365-294X.2005.02553.x 15969739

[B30] Jakobsson M Rosenberg NA CLUMPP: a cluster matching and permutation program for dealing with label switching and multimodality in analysis of population structure Bioinformatics 2007 23 1801 1806 10.1093/bioinformatics/btm233 17485429

[B31] Rosenberg NA Distruct: a program for the graphical display of population structure Mol Ecol Notes 2004 4 137 138

[B32] Conrad DF Jakobsson M Coop G Wen X Wall JD Rosenberg NA Pritchard JK A worldwide survey of haplotype variation and linkage disequilibrium in the human genome Nat Genet 2006 38 11 1251 1260 10.1038/ng1911 17057719

[B33] Nielsen R Estimation of population parameters and recombination rates from single nucleotide polymorphisms Genetics 2000 154 2 931 942 10655242 10.1093/genetics/154.2.931 PMC1460954

[B34] Peleg Z Saranga Y Suprunova T Ronin Y Röder MS Kilian A Korol AB Fahima T High-density genetic map of durum wheat x wild emmer wheat based on SSR and DArT markers Theor Appl Genet 2008 117 1 103 115 10.1007/s00122-008-0756-9 18437346

[B35] Würschum T Langer SM Longin CF Korzun V Akhunov E Ebmeyer E Schachschneider R Schacht J Kazman E Reif JC Population structure, genetic diversity and linkage disequilibrium in elite winter wheat assessed with SNP and SSR markers Theor Appl Genet 2013 126 1477 1486 10.1007/s00122-013-2065-1 23429904

[B36] Zhang D Bai G Zhu C Yu J Carver BF Genetic diversity, population structure, and linkage disequilibrium in U.S. elite winter wheat Plant Genome 2010 3 117 127 10.3835/plantgenome2010.03.0004

[B37] Chao S Dubcovsky J Dvorak J Luo MC Baenziger SP Matnyazov R Clark DR Talbert LE Anderson JA Dreisigacker S Glover K Chen J Campbell K Bruckner PL Rudd JC Haley S Carver BF Perry S Sorrells ME Akhunov ED Population- and genome-specific patterns of linkage disequilibrium and SNP variation in spring and winter wheat (Triticum aestivum L.) BMC Genomics 2010 11 727 10.1186/1471-2164-11-727 21190581 PMC3020227

[B38] Chen X Min D Yasir TA Hu YG Genetic diversity, population structure and linkage disequilibrium in elite Chinese winter wheat investigated with SSR markers PLoS ONE 2012 7 9 e44510 10.1371/journal.pone.0044510 22957076 PMC3434133

[B39] Maccaferri M Sanguineti MC Demontis A El-Ahmed A Garcia del Moral L Maalouf F Nachit M Nserallah N Ouabbou H Rhouma S Royo C Villegas D Tuberosa R Association mapping in durum wheat grown across a broad range of water regimes J Exp Bot 2011 62 2 409 438 10.1093/jxb/erq287 21041372

[B40] Maccaferri M Sanguineti MC Noli E Tuberosa R Population structure and long-range linkage disequilibrium in a durum wheat elite collection Mol Breed 2005 15 271 290 10.1007/s11032-004-7012-z

[B41] Somers DJ Banks T Depauw R Fox S Clarke J Pozniak C McCartney C Genome-wide linkage disequilibrium analysis in bread wheat and durum wheat Genome 2007 50 6 557 567 10.1139/G07-031 17632577

[B42] Van Inghelandt D Melchinger AE Lebreton C Stich B Population structure and genetic diversity in a commercial maize breeding program assessed with SSR and SNP markers Theor Appl Genet 2010 120 1289 1299 10.1007/s00122-009-1256-2 20063144 PMC2854351

[B43] Haasl RJ Payseur BA Multi-locus inference of population structure: a comparison between single nucleotide polymorphisms and microsatellites Heredity 2011 106 158 171 10.1038/hdy.2010.21 20332809 PMC2892635

