# Supplementary figures and images for: Wheat in the Mediterranean revisited – tetraploid wheat landraces assessed with elite bread wheat Single Nucleotide Polymorphism markers
Source: BMC Genet. 2014 May 8;15:54. doi: 10.1186/1471-2156-15-54 (PMC4029936; doi:10.1186/1471-2156-15-54)

a

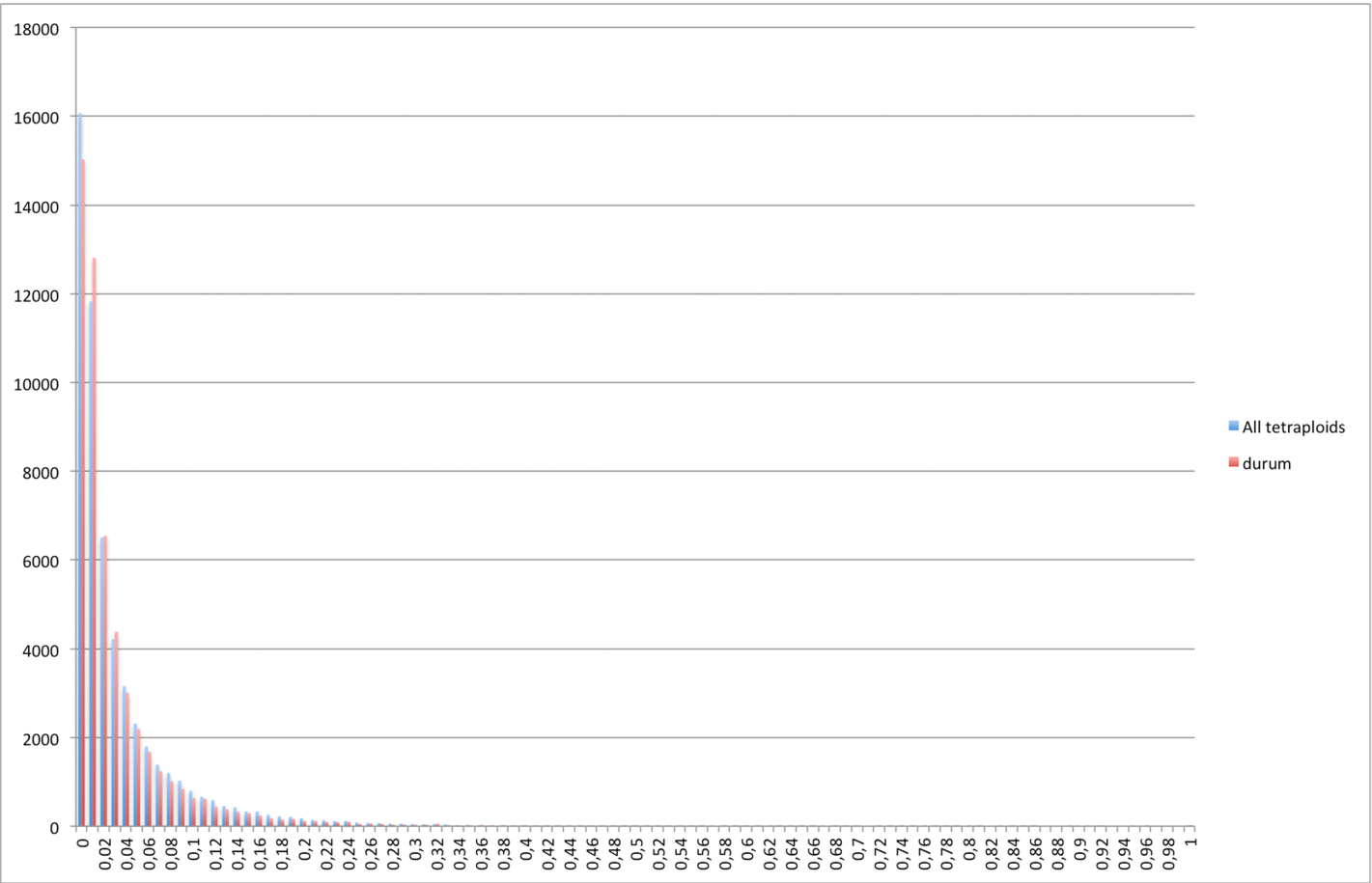

b

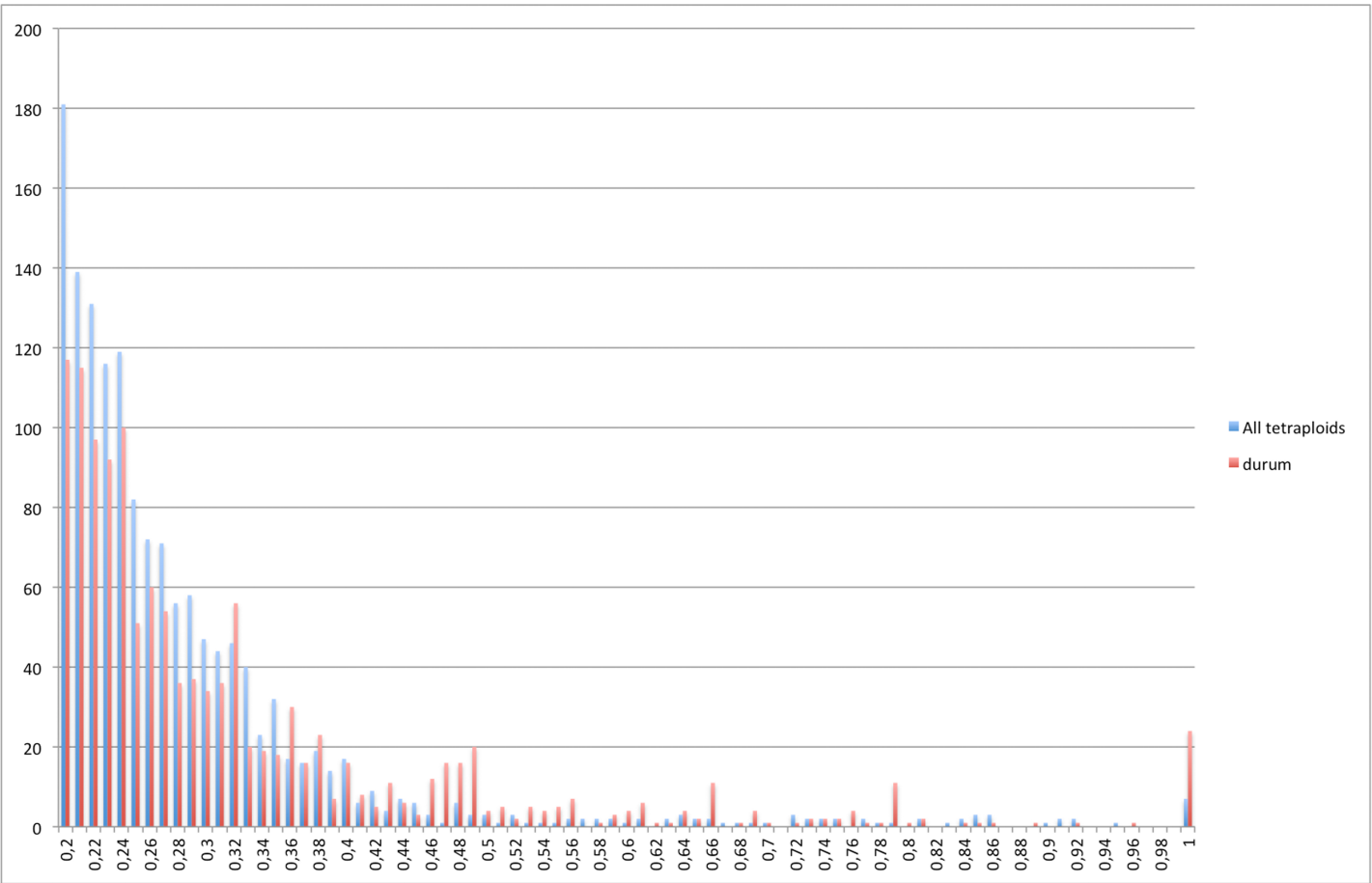

Supplement: Additional file 4 — Distribution of linkage disequilibrium values calculated between pairs of loci located on different chromosomes. a) All values of r2, b) Values of r2 from 0.2 and higher. [file 1471-2156-15-54-S4.pdf]

**a**

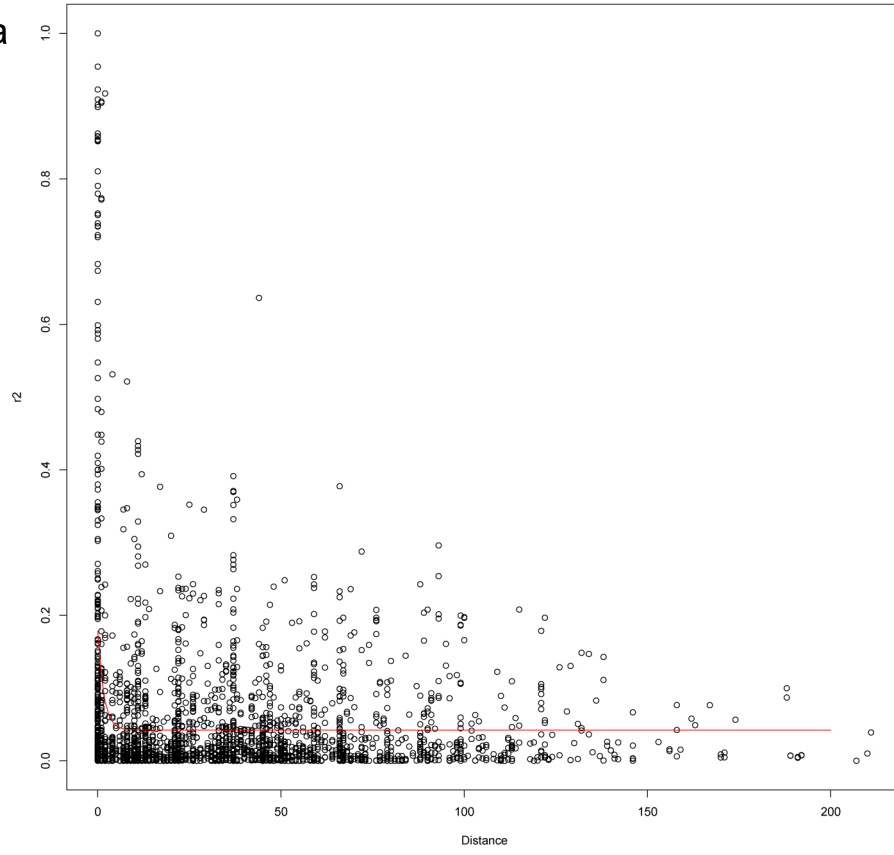

**b**

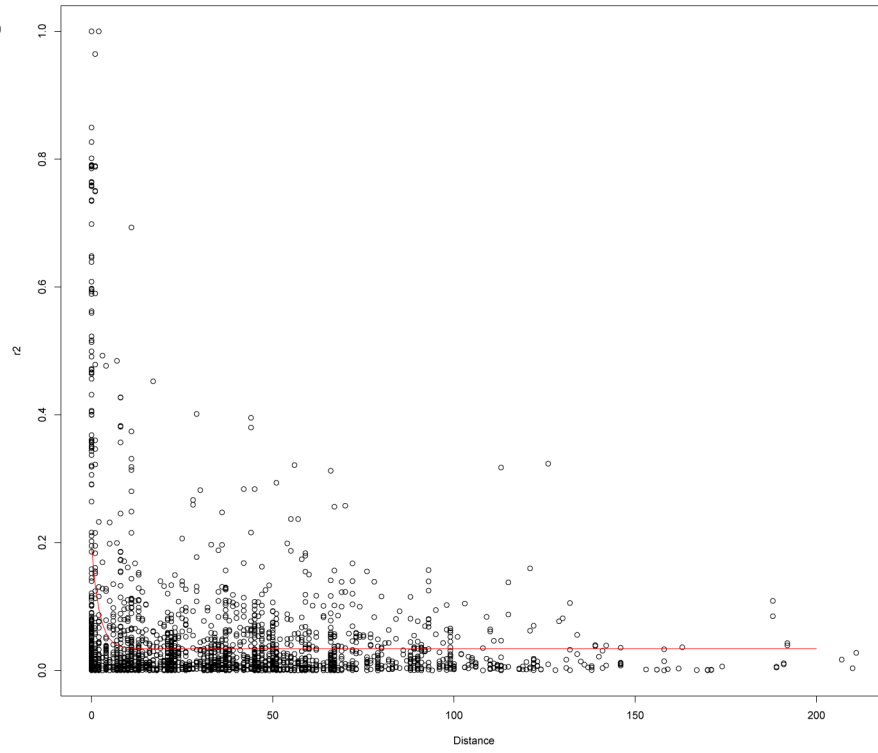

Supplement: Additional file 5 — Linkage disequilibrium (r 2 ) between linked markers plotted against genetic distance with a non-linear regression line fitted to the values. a) All tetraploid wheats; b) all durum landraces. [file 1471-2156-15-54-S5.pdf]

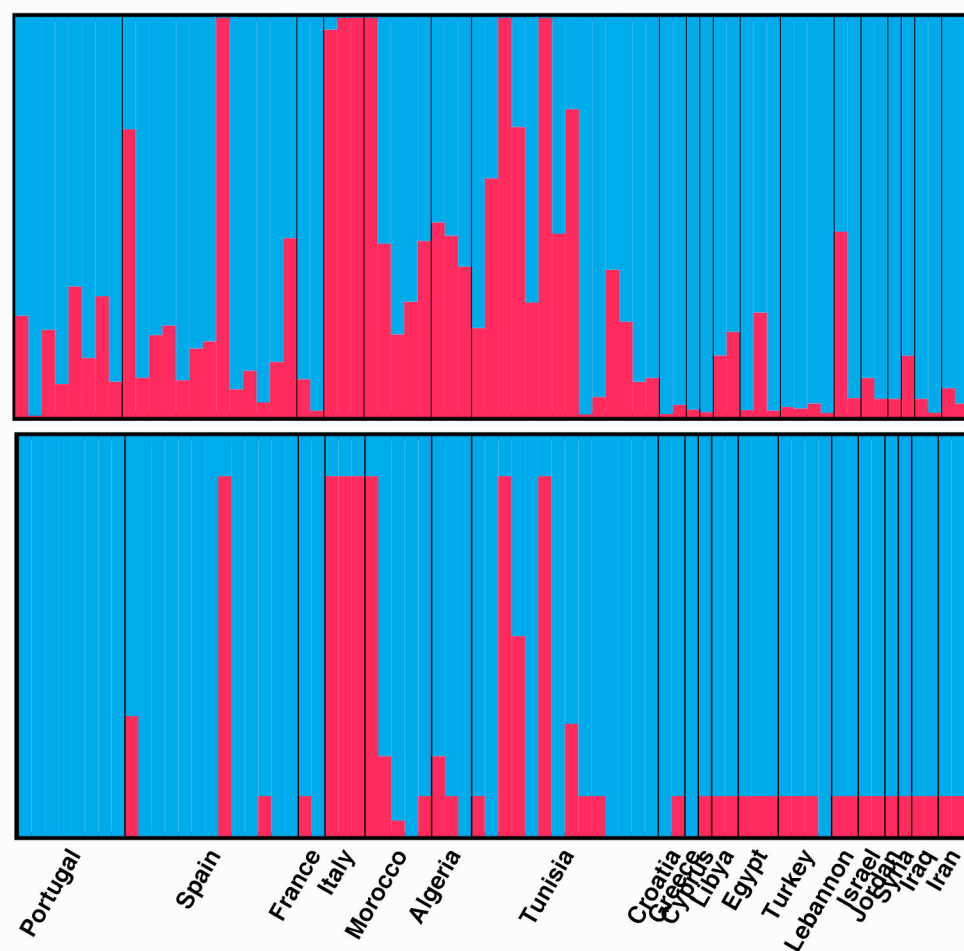

A

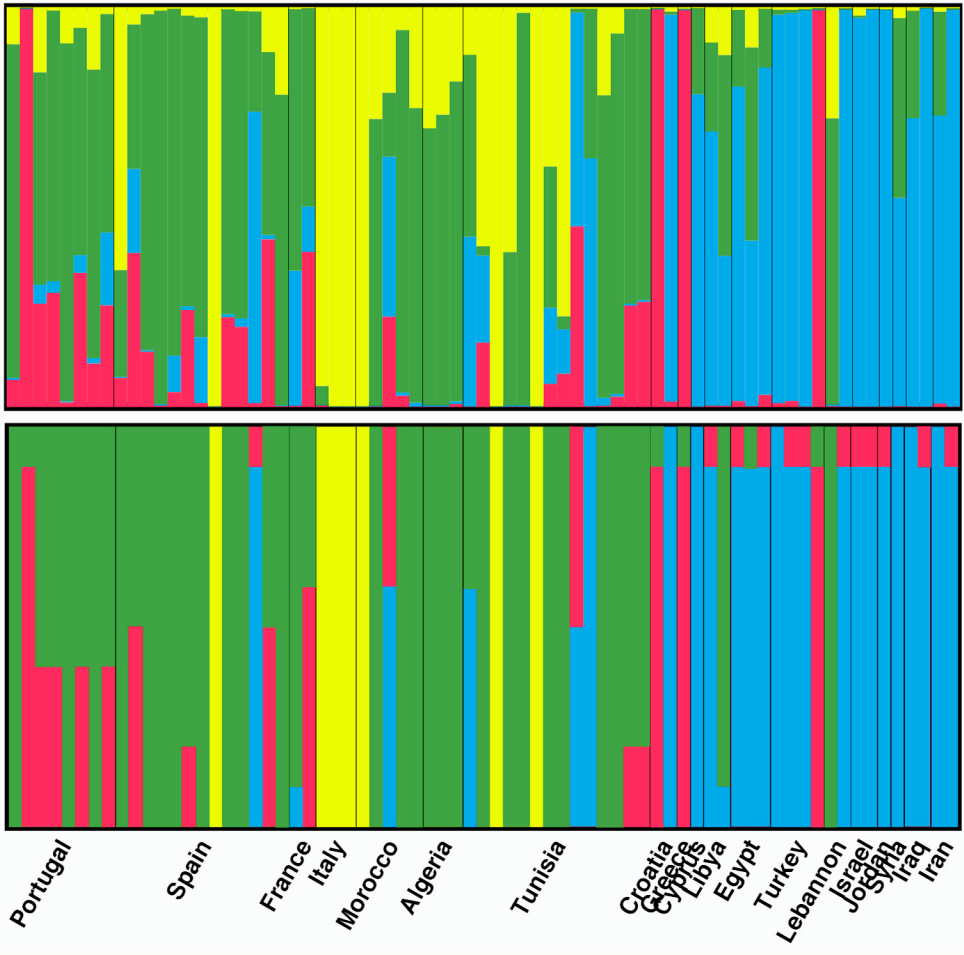

*B*

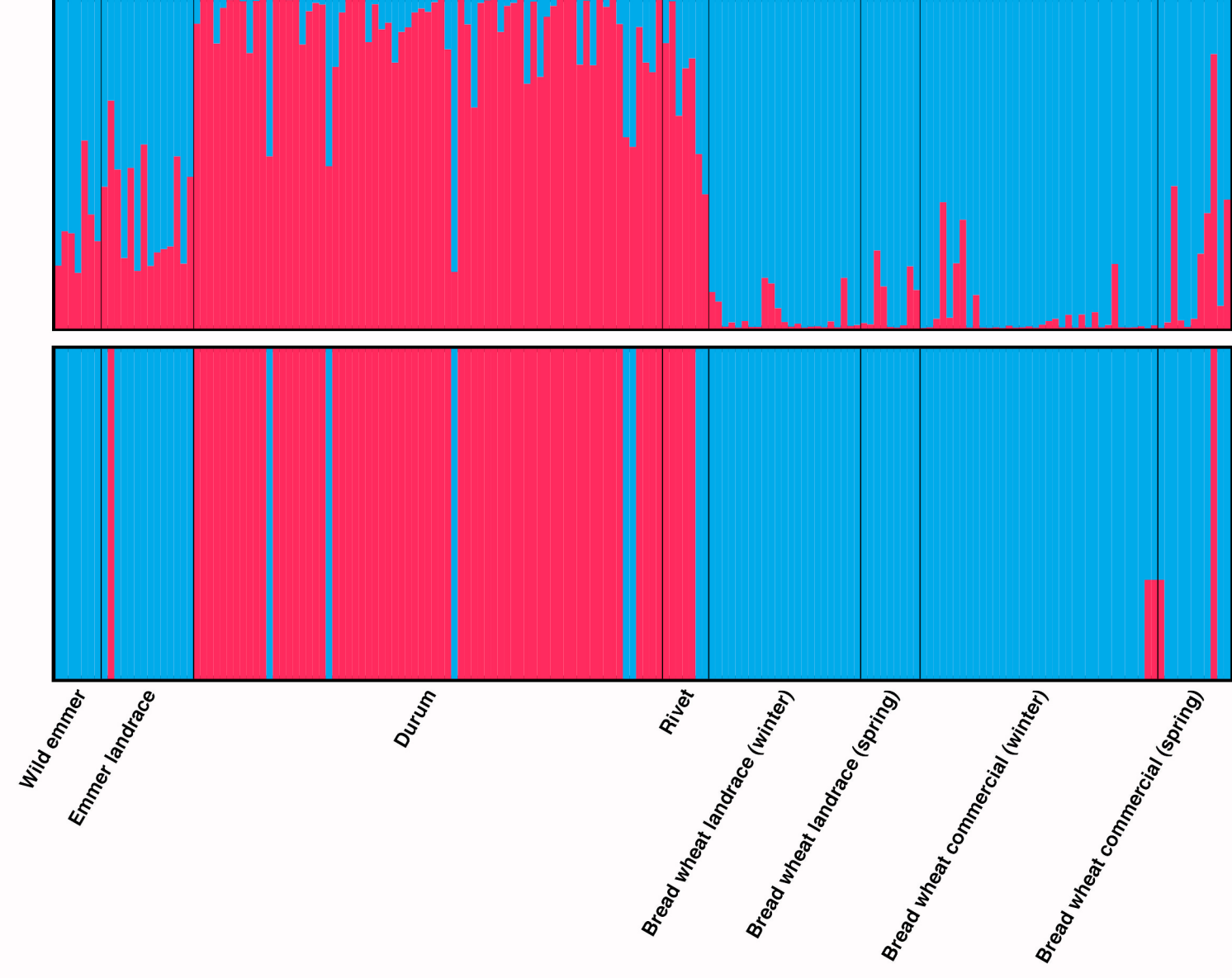

C

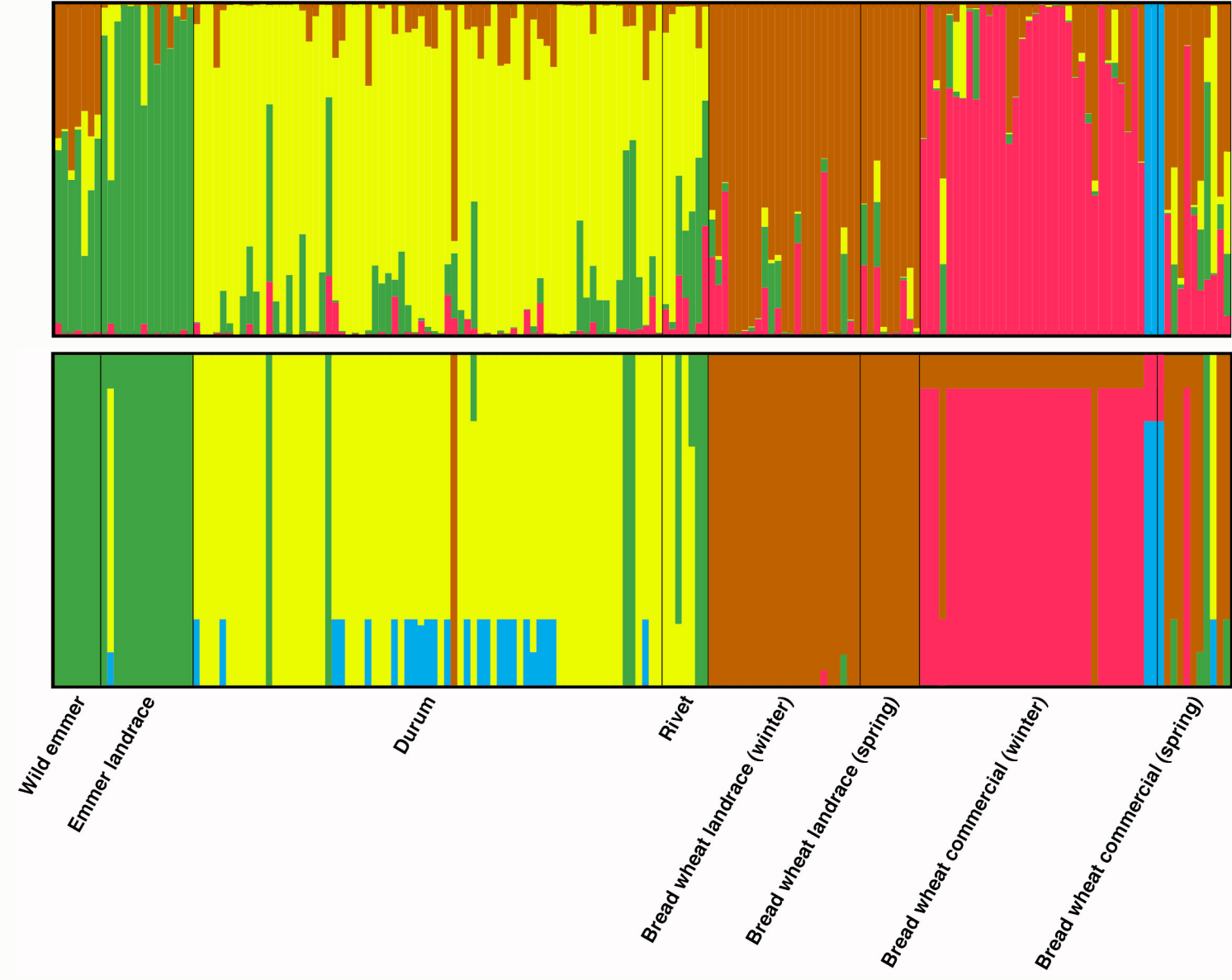

*D*

Supplement: Additional file 6 — Results of Structure analysis of wheat accessions based on 369 SNPs using the “ admixture ” option (top panel) and the “ no admixture ” option (bottom panel) for a) K = 2 model with the durum accessions; b) K = 4 model with durum accessions; c) K = 2 model with the complete set of accessions; d) K = 5 model with the complete set of accessions. [file 1471-2156-15-54-S6.pdf]

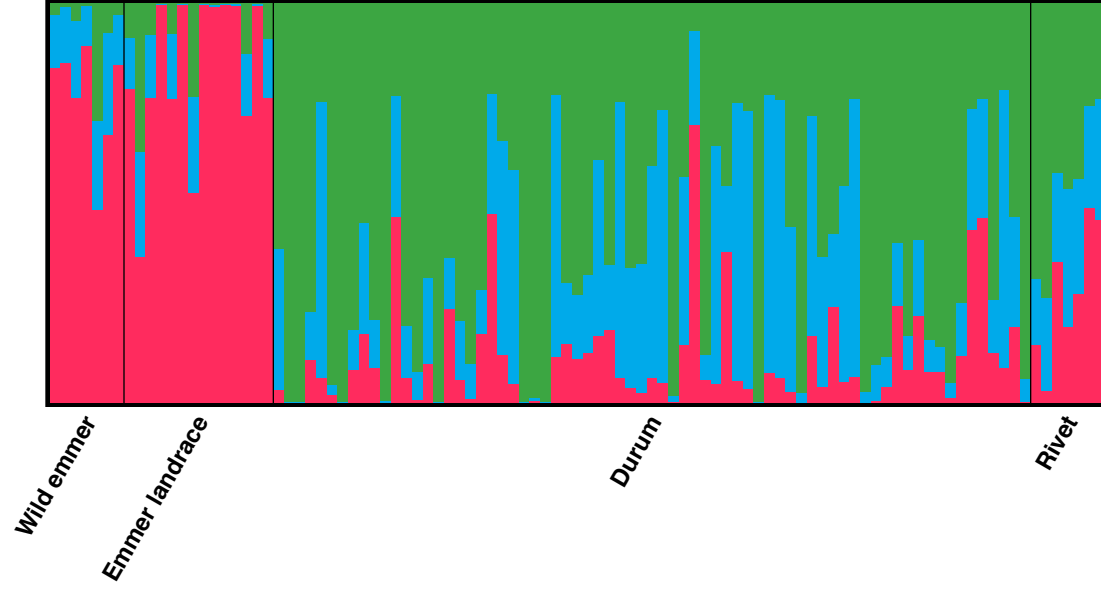

Supplement: Additional file 7 — Results of Structure analysis of the K = 3 model for the tetraploid wheat set. [file 1471-2156-15-54-S7.pdf]

a

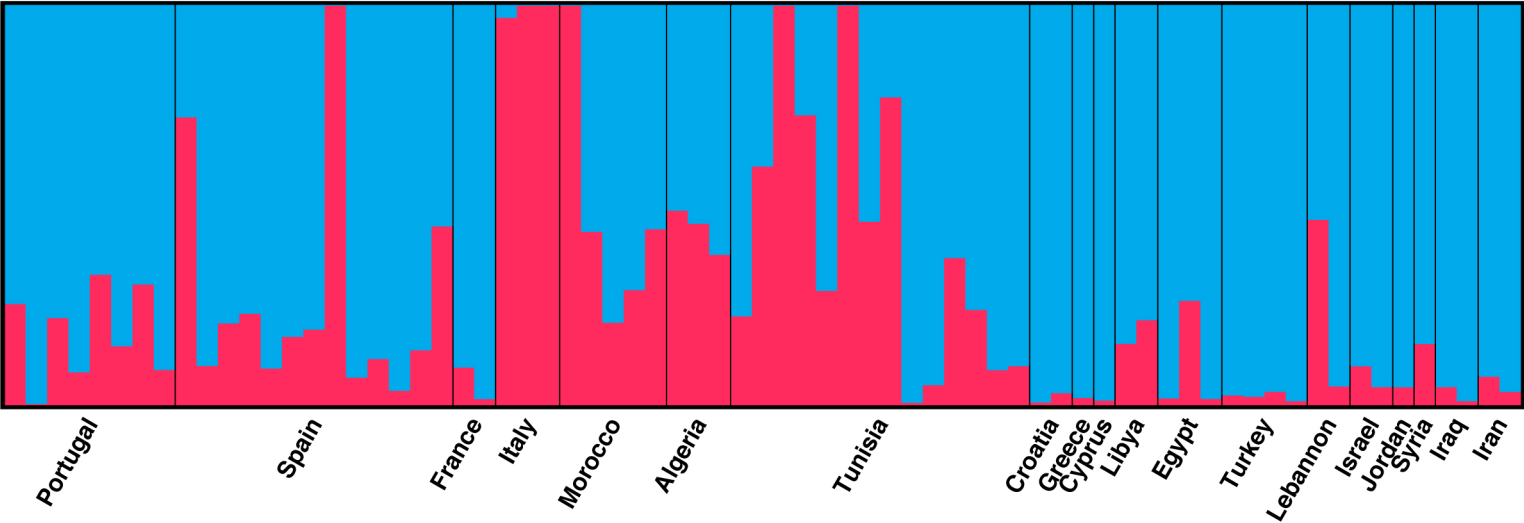

b

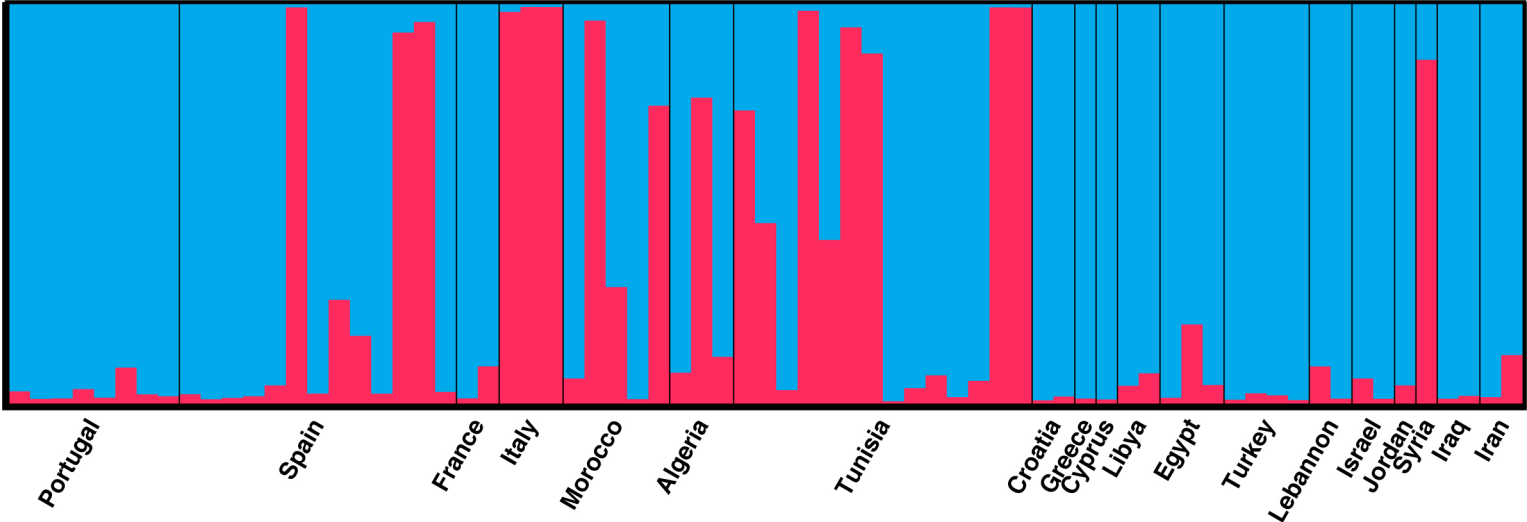

Supplement: Additional file 8 — Results of Structure analysis of the set of durum accessions for the K = 2 model based on a) 369 SNPs and b) 29 SSRs respectively. [file 1471-2156-15-54-S8.pdf]

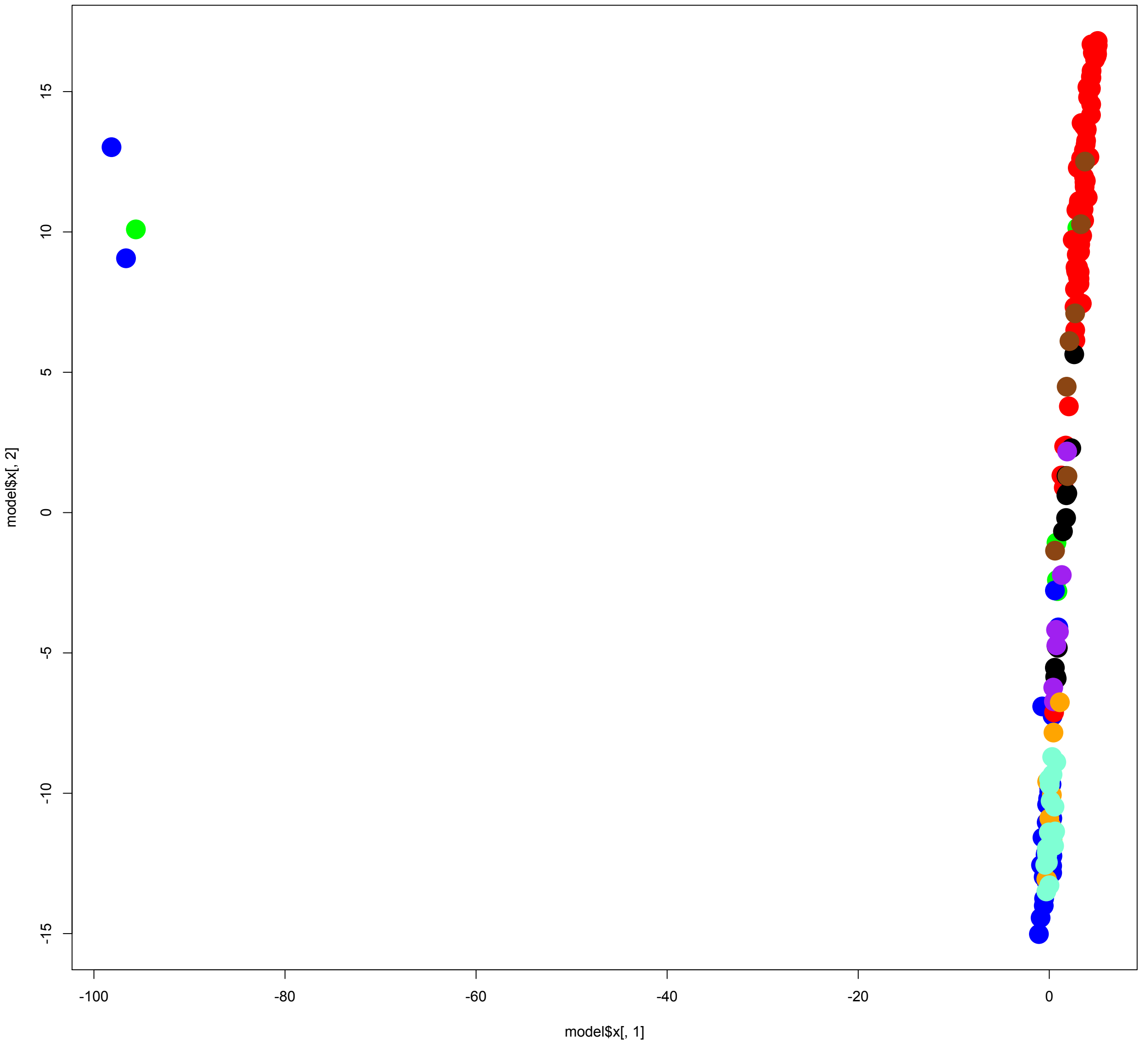

Supplement: Additional file 9 — Results of Principal Component Analysis of the complete set of wheat accessions. The distinctiveness of Yumai 34, Anahuac 75 and Ukrainka is evident (top left corner). Black = wild emmer; purple = landrace emmer; red = durum; orange = rivet; blue = landrace bread wheat; green = commercial bread wheat. [file 1471-2156-15-54-S9.pdf]

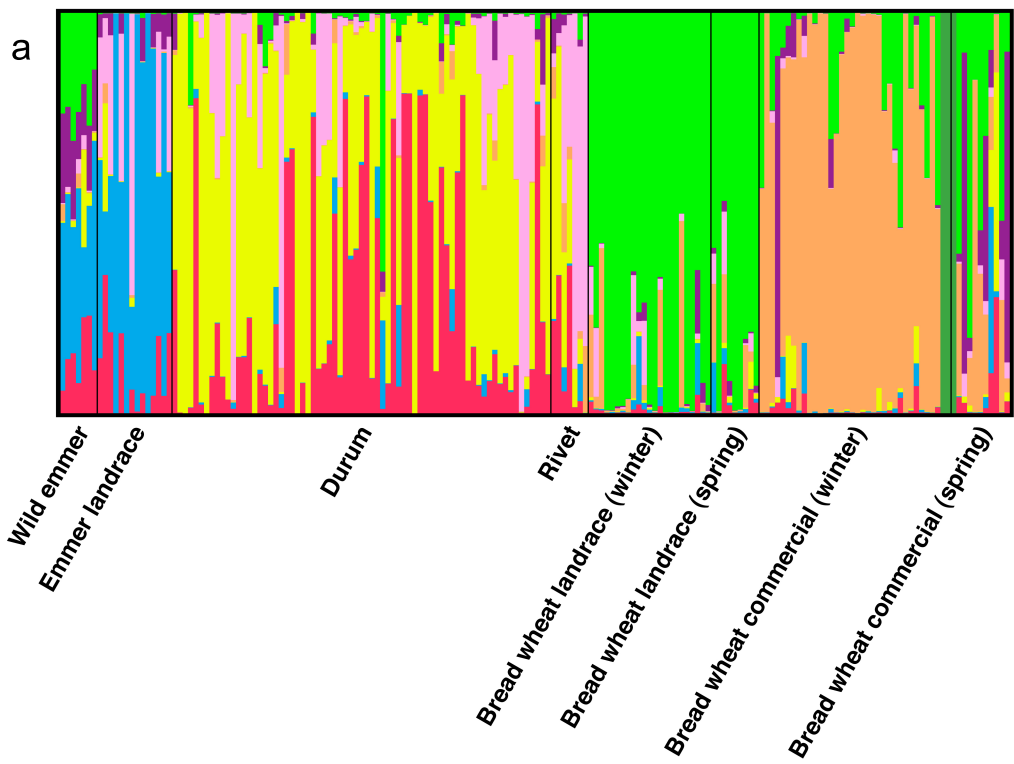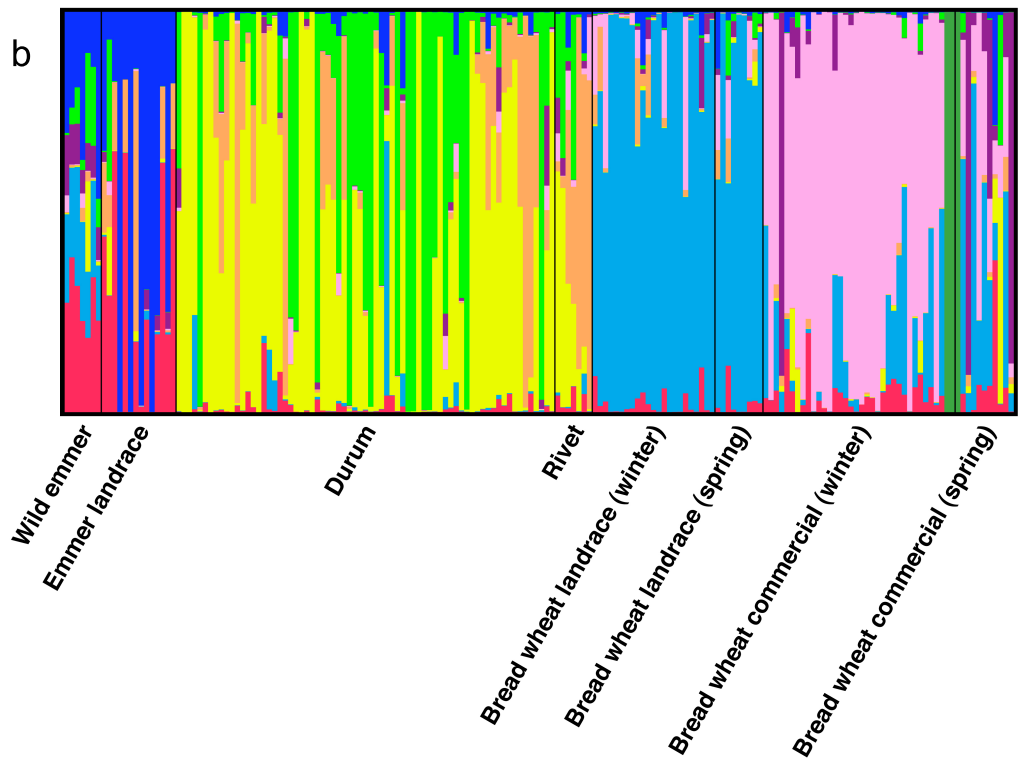

Supplement: Additional file 10 — Results of Structure analysis of the complete set of wheat accessions for the a) K = 8 model and b) K = 9 model. [file 1471-2156-15-54-S10.pdf]
